# Activation of PI3K/Akt pathway by G protein‐coupled receptor 37 promotes resistance to cisplatin‐induced apoptosis in non‐small cell lung cancer

**DOI:** 10.1002/cam4.6543

**Published:** 2023-09-21

**Authors:** Han Liu, Yingjie Zhu, Huikun Niu, Jing Jie, Shucheng Hua, Xiaoxue Bai, Shuai Wang, Lei Song

**Affiliations:** ^1^ Department of Respiratory Medicine The First Hospital of Jilin University Changchun Jilin China; ^2^ Department of Respiratory and Critical Care Medicine The Second Affiliated Hospital of Fujian Medical University Quanzhou Fujian China; ^3^ Department of General Practice The First Hospital of Jilin University Changchun Jilin China; ^4^ Department of Vascular Surgery, General Surgery Center The First Hospital of Jilin University Changchun Jilin China

**Keywords:** cisplatin, EMT, GPR37, NSCLC, PI3K/Akt pathway

## Abstract

**Objectives:**

Lung cancer is a major public health concern and represents the most common cause of cancer‐related death worldwide. Among eukaryotes, the G protein‐coupled receptor (GPCR) family stands as the largest group of membrane proteins. Alterations in GPCR gene expression and dysregulation of signal transduction have been recognized as the markers of malignancy. As a member of the GPCR family, G protein‐coupled receptor 37 (GPR37) exhibits unknown functions in tumors, particularly in non‐small‐cell lung cancer (NSCLC)

**Methods:**

We explored the expression and prognosis of GPR37 in NSCLC through TCGA, GTEx, GEO, and GEPIA2. We detected the expression of GPR37 in NSCLC tissues and cell lines. The study explored the influence of GPR37 on tumor cell proliferation. Furthermore, we examined the effects of GPR37 on tumor cell apoptosis and invasion. Most importantly, we investigated whether GPR37 affects cisplatin‐induced drug resistance in NSCLC. Furthermore, by conducting animal experiments, we assessed the impact of GPR37 on NSCLC and delved into underlying mechanisms.

**Results:**

(1) In NSCLC, the expression of GPR37 is markedly higher than that in corresponding normal tissues. We found that elevated GPR37 expression predicts an unfavorable prognosis. (2) It was demonstrated that GPR37 positively regulates NSCLC cell invasion, migration, and proliferation, suppresses cell apoptosis, heightens resistance to cisplatin, and promotes tumor formation and growth. Conversely, we observed that GPR37 knockdown suppresses NSCLC cell invasion, migration, and proliferation, promotes cell apoptosis, increases sensitivity to cisplatin, and affects tumor formation and growth. (3) GPR37 activates PI3K/Akt/mTOR signal transduction pathways to mediate epithelial‐mesenchymal transition (EMT), thereby promoting the progression of NSCLC.

**Conclusions:**

It was suggested that GPR37 acts a crucial role in promoting the occurrence and development of NSCLC. Knockdown of GPR37 significantly inhibits the occurrence and development of NSCLC. Therefore, our findings demonstrated that GPR37 may represent a viable therapeutic target for NSCLC.

## 1 INTRODUCTION

Cancer, the foremost reason for mortality worldwide, is one of the important obstacles of the extension of human life expectancy. In 2021, World Health Organization (WHO) data show that lung cancer is the most common cause of cancer‐related deaths in 60 countries and the second largest malignant tumor worldwide. Despite significant advancements in medical technology and therapeutic strategies, the 5‐year survival rate of patients afflicted with non‐small cell lung cancer (NSCLC) stands at an approximate 20%.^1^ While the combination of platinum drugs and tyrosine kinase inhibitors has emerged as the quintessential initial chemotherapy approach for NSCLC,^2^, ^3^ a significant proportion of patients succumb to tumor resistance after undergoing six courses of treatment. The exact etiology of this therapeutic impediment, however, remains poorly understood.^4^ Currently, chemoresistance, a multifactorial phenomenon, is associated with the rapid drug metabolism, dysregulation of cellular drug uptake and detoxification, and high DNA repair, in which dysregulated proapoptotic and anti‐apoptotic pathways play a key role.^5^ Lung cancer was considered to become a major threat to health and a prominent social burden in the next 20 years. Therefore, elucidating the molecular mechanisms of lung cancer development and chemical resistance, looking for possible biomarkers considering genetics, and determining effective treatment methods can provide new evidence‐based medical evidence and strategies for precision medicine for lung cancer.

G protein‐coupled receptors (GPCRs) constitute the most extensive family of membrane proteins within eukaryotes. Altered GPCR gene expression and dysregulated signal transduction are recognized hallmarks of malignancy.^6^ The first GPCR was discovered in 1986 when researchers found that it binds to angiotensin and exerts vasodilator and antiproliferative effects, and it was identified as MAS protein.^7^ Structurally, GPCRs share a typical architecture comprising an intracellular domain and an extracellular domain, a transmembrane domain stabilized by seven alpha helix structures.^8^ Collectively, they can respond to various stimuli, including ions, odorants, neurotransmitters, chemokines, and light,^9^ which structurally switch receptor proteins into an active state.^10^ The GPCR protein family has received extensive attention because of its potential therapeutic targets in many pathological conditions. Moreover, one‐third or even one‐half of the clinically marketed drugs exert their effects by binding to GPCRs. G protein‐coupled receptor 37 (GPR37) is also known as Parkinson‐associated endothelin receptor‐like receptor. At present, GPR37 is considered by most scholars as an orphan receptor, which lacks endogenous ligands to trigger GPCR signaling, hindering the study of its function.^11^ Wang et al. presented that gastric cancer might be promoted by GPR37.^12^ By contrast, GPR37 is underexpressed in hepatocellular carcinoma^13^ and multiple myeloma cell adhesion models.^14^ Therefore, GPR37 poses a double‐edged sword in malignancy. In recent years, the research of GPR37 in lung cancer has also made new progress. Wang et al. found that GPR37 is a potential oncogene of LUAD, and its promoting effects on the malignant progression of LUAD may be realized via TGF‐β/Smad pathway.^15^ Xie et al. found that GPR37 promotes cancer growth by binding to CDK6 and represents a new theranostic target in lung adenocarcinoma.^16^ Nevertheless, the function of GPR37 in NSCLC remains generally obscure and warrants further investigation.

In the early 1980s, a correlation between cancer and epithelial‐mesenchymal transition (EMT) was reported. EMT is a ubiquitous process that characterizes the vast majority of tumors during their progressive stage; thus, cancers of epithelial origin are determined by EMT processes.^17^ After EMT activation, tumor epithelial cells forfeit their cell polarity and the ability to maintain intercellular adhesion while gaining migratory and invasive attributes characteristic of mesenchymal cells.^17^ Evidence highlights the impact of EMT on the pathogenesis of several types of malignancies, including but not limited to prostate, lung, liver, pancreas, and breast cancer.^18^, ^19^ In EMT, cells get hold of the characteristics of mesenchymal cells. Mesenchymal biomarkers such as fibronectin, vimentin, and N‐cadherin were significantly upregulated, and these mesenchymal markers are closely associated with cell invasion and related to the malignant phenotype of NSCLC.^20^, ^21^, ^22^

Moreover, the PI3K/Akt/mTOR pathway acts a crucial role in modulating cell growth and metabolism. The relevance of PI3K in oncogenesis was initially postulated in 1985 and has since been well‐documented.^23^ PI3Ks constitute a family of intracellular lipid kinases that may be categorized into three distinct classes (I‐III), each performing a unique role in signal transduction. Specifically, class I PI3Ks assume an important role in this context and they are differentiated into two subclasses: class IA PI3Ks, triggered by growth factor receptor tyrosine kinase, and class IA PI3Ks, triggered by GPCRS.^24^ As a member of the PKA/PKG/PKC protein kinase family, Akt activation subsequently induces several potential downstream effects. By contrast, the activated nuclear factor kappa‐B (NF‐κB) can modulate the expression of a series of genes related to differentiation, apoptosis, cell adhesion, cell survival, immune regulation, and cell cycle control.^25^ Additionally, activation of the mechanistic target of mTOR protein kinase represents another consequential downstream process initiated by Akt activation. It is triggered by TSC2 phosphorylation, which in turn activates Rheb, leading to the activation of mTORC1, a multiprotein complex. mTORC1, in turn, propels downstream activation of the eIF4 complex and subsequently inhibits apoptosis, regulates cell cycle, and promotes tumorigenesis.^26^

Through the screening of the Cancer Genome Atlas (TCGA) database, we found that the expression of GPR37 shows a marked increase in patients afflicted with NSCLC and appears to exert a discernible influence upon disease prognosis. Based on the analysis of clinical data at our center, we also obtained similar findings, that is, the expression of GPR37 in para‐cancerous tissues of NSCLC was lower than that in tumor. Moreover, the study demonstrated that the expression of GPR37 was lower in normal alveolar epithelial cells than that in different tumor cell lines. GPR37 can drive the invasion, migration and proliferation of tumor cells, and has a certain anti‐apoptotic effect. GPR37 is able to elevate the resistance of platinum‐based chemotherapy drugs and promote EMT in NSCLC and activate PI3K/Akt/mTOR signal transduction pathways. GPR37 knockdown can inhibit EMT in NSCLC and affect the activation of PI3K/Akt/mTOR signal transduction pathways. Consequently, further in‐depth research on GPR37 will not only help reveal the development mechanism and tumor drug resistance mechanism of NSCLC but also use GPCR as a drug target to provide a theoretical basis for individualized treatment options for NSCLC.

## 2 MATERIALS AND METHODS

### 2.1 Bioinformatics analysis

We used the database information in TCGA, applied the online analysis tool TIMER2.0, and entered GPR37 in the Cancer Exploration module to compare the expression of GPR37 between normal tissues and tumors. Information obtained from TCGA encompassed 501 samples of lung squamous cell carcinoma (LUSC) with 51 samples of normal control tissues, 515 samples of lung adenocarcinoma (LUAD) with 59 samples of normal control tissues. Moreover, the Gene Expression Profiling Interactive Analysis 2 (GEPIA2) was progressed to select the patient information from TCGA and GTEx databases, and the differential analysis of GPR37 expression was performed by statistically analyzing the data in LUSC and LUAD tumor, and normal control tissues. We also analyzed the NSCLC dataset of NCBI‐GEO database to investigate the differential expression of GPR37 in NSCLC samples and normal samples. We obtained gene expression profiles of GSE18842,^27^ GSE74706,^28^ and GSE21933^29^ in lung cancer and normal lung tissues. Kyoto Encyclopedia of Genes and Genomes (KEGG) pathway enrichment analysis of co‐expressed genes was performed by cluster Profiler package^30^ of R software, and visual analysis of data was performed by ggplot2 software package.

The Survival Analysis module of GEPIA2 was applied to evaluate the overall survival (OS) of GPR37. The GEPIA platform implements the log‐rank test for hypothesis evaluation, with the inclusion of 95% confidence interval findings in its outcomes.

### 2.2 Patient recruitment and lung tissue specimens

In this study, 20 patients with a histologically confirmed diagnosis of NSCLC who underwent surgery were recruited. The lung cancer and adjacent tissues of these participants were collected. The admission criteria were as follows: (1) age ≥18 years, (2) definite diagnosis of NSCLC by postoperative histopathology, and (3) no history of other tumors. The exclusion criteria were as follows: (1) chemotherapy and radiotherapy for other diseases, (2) history of other malignant tumors, and (3) diseases with immunodeficiency. Specimens were collected and then cryopreserved in liquid nitrogen for storage at −80°C until RNA extraction was performed.

All participants provided informed consent before enrolling in this study, which was endorsed by the Ethics Committee of the First Hospital of Jilin University (2021‐170).

### 2.3 Preparation of human lung tissue sections

Sampling and fixing, dehydration, immersion in wax, embedding, sectioning, and dewaxing were performed. For hematoxylin and eosin (HE) staining of tissue sections, HE staining, eosin staining, dehydrating sections, transparent sectioning, and mounting were performed.

### 2.4 Immunohistochemistry (IHC) staining

To prepare human lung tissue slices, the sodium citrate buffer was added, boiled, fixed, and washed, and then, an endogenous peroxidase‐blocking agent was added. After washing, a nonspecific staining blocker was added. After drying, the GPR37 polyclonal antibody was added dropwise as the primary antibody (diluted 1:200, Invitrogen). The primary antibody was removed, and a secondary antibody of goat anti‐rabbit polymer (diluted 1:200, ABclonal) was subsequently introduced. Streptavidin‐peroxidase was utilized, and the specimen was then incubated at room temperature. Thereafter, DAB chromogenic solution was added and then soaked in hematoxylin for counterstaining, and the slides were dehydrated in gradient alcohol and then soaked in xylene. Mount the slides for observation under a microscope.

### 2.5 Cell line culture

Frozen Mrc‐5, BEAS‐2B, CCD‐19Lu, A549, H1299, Calu‐1, Calu‐3, H460, and HCC827 cells were taken from −80°C liquid nitrogen storage and immediately placed in a 37°C water bath. BEAS‐2B, CCD‐19Lu, A549, H1299, and H460 cells were cultured in Dulbecco's modified eagle medium (Gibco), Mrc‐5, Calu‐3, and Calu‐1 cells were cultured in minimum essential medium (Invitrogen), while HCC827 cells were cultured in RPMI 1640 (Gibco). All cells were cultured under standard conditions, which involved incubating them at 37°C in a 5% CO_2_. According to different requirements, 10% fetal bovine serum (Biosharp), penicillin–streptomycin liquid (HyClone), and Hepes (Solarbio) were added to the culture medium.

### 2.6 Western blot assay

For the extraction of total tissue protein, human lung tumor tissue and matched para‐cancerous tissue were taken out from −80°C liquid nitrogen storage. The tissues were shredded, thoroughly grounded, and added with radioimmunoprecipitation assay (Beyotime, China) to fully lyse it. Then, the sample was supplemented with precooled phosphate‐buffered saline (PBS, Biosharp), following which it was resuspended and subjected to centrifugation. The resulting supernatant was then collected via aspiration.

Extraction of total cell protein: The original culture medium was removed from the cell culture dish, washed with PBS, and then added with an appropriate amount of lysate (the amount of lysate depends on the size of the culture dish) until the lysate completely covered the cells and fully lysed. The lysate and cell debris were transferred to an Eppendorf tube and centrifuged, and the supernatant was aspirated.

The bicinchoninic acid protein concentration assay kit (Biosharp) was utilized to calculate the protein content. The protein content was transferred to polyvinylidene difluoride membranes and subsequently obstructed with 5% nonfat dry milk for 60 minutes. Once obstructed, the membrane was incubated with primary antibodies. The primary antibodies include GPR37 (diluted 1:200, Invitrogen), GAPDH (diluted 1:10000, ABclonal), vimentin (diluted 1:500, ABclonal), N‐cadherin (diluted 1:500, ABclonal), E‐cadherin (diluted 1:500, ABclonal), P‐AKT1 (diluted 1:500, ABclonal), P‐EIF4EBP1 (diluted 1:2000, ABclonal), p70S6K (diluted 1:500, ABclonal), P‐p70S6K (diluted 1:100, ABclonal), mTOR (diluted 1:500, ABclonal), P‐mTOR (diluted 1:500, ABclonal), PI3K (diluted 1:500, ABclonal), P‐PI3K (diluted 1:500, ABclonal), EIF4EBP1 (diluted 1:500, ABclonal), and AKT1 (diluted 1:500, ABclonal). The secondary antibodies were HRP goat anti‐rabbit IgG and HRP goat anti‐mouse IgG (diluted 1:2000, ABclonal).

### 2.7 RNA extraction and quantitative RT‐PCR (qRT‐PCR)

Total RNA was extracted from lung cancer tissues, para‐cancerous tissues, and cells via Trizol reagent (Invitrogen). Using a TransStart Top Green qPCR SuperMix Kit (TransGen), the extracted RNA was reverse‐transcribed into cDNA. Then, Real‐time PCR System (Applied Biosystems) was carried out for qRT‐PCR. The expressions of GPR37 mRNA and GAPDH mRNA were determined through qRT‐PCR. qPCR data were normalized according to 2−ΔΔCt method, which facilitates the analysis of relative gene expression in qRT‐PCR experiments.

### 2.8 siRNA interference and plasmid transfection

The GPR37‐siRNA sequences were as follows: GPR37‐shRNA‐1F, CCGGCGAGGGAATAAACGGCAGATTCTCGAGAATCTGCCGTTTATTCCCTCGTTTTT; GPR37‐shRNA‐1R, AATTAAAAACGAGGGAATAAACGGCAGATTCTCGAGAATCTGCCGTTTATTCCCTCG; GPR37‐shRNA‐2F, CCGGCCTTAATATCATCAGCCAGTTCTCGAGAACTGGCTGATGATATTAAGGTTTTT; GPR37‐shRNA‐2R, AATTAAAAACCTTAATATCATCAGCCAGTTCTCGAGAACTGGCTGATGATATTAAGG; Mock‐shRNA‐F, CCGGCAACAAGATGAAGAGCACCAACTCGAGTTGGTGCTCTTCATCTTGTTGTTTTTG; Mock‐shRNA‐R, AATTCAAAAACAACAAGATGAAGAGCACCAACTCGAGTTGGTGCTCTTCATCTTGTTG. Plasmids were extracted based on the instructions of the plasmid extraction kit (Tiangen, China). Transfection was performed using Lipofectamine 3000 (Invitrogen). In this study, the lentivirus packaging plasmid with GPR37 overexpression and knockdown were constructed and transfected into H1299, H460, and A549 cells to obtain stable expressions of GPR37 overexpression (GPR37 group), empty vector group (vector group), GPR37 knockdown group (shGPR37 group), and GPR37 knockdown control group (sh‐mock group).

### 2.9 Cell proliferation assay

To evaluate cell proliferation, three distinct assays were employed: the colony formation assay, the 5‐ethynyl‐2′‐deoxyuridine (EdU) Cell Proliferation Kit (Invitrogen), and the Cell Counting Kit‐8 (CCK‐8, APExBIO) assay. For conducting the CCK‐8 assay, 4000 cells per well were seeded onto 96‐well plates and thereafter cultivated for a period of 24 h. Subsequently, 10 μL of CCK‐8 reagent was added to each well at 24, 48, 72, and 96 h, and the plate was left to incubate for 2 h in a 37°C incubator. Then, the absorbance at 450 nm was quantified through a microplate reader. For the EdU assay, NSCLC cells were seeded into 6‐well culture plates and analyzed based on the kit instructions. For the colony formation assay, cells in different groups were taken for digestion, blown repeatedly to form a single cell, and prepared into a cell suspension. Then, 200 cells per well were added to a 6‐well plate and placed in an incubator. After culturing for 2 weeks, formaldehyde was added for fixation and then stained with 0.1% crystal violet.

### 2.10 Flow cytometry

Detection of apoptosis was executed by implementing the Cell Cycle and Apoptosis Analysis Kit (Beyotime). All procedures were progressed according to the manufacturer's instructions. Cell apoptosis was detected using the DxFLEX flow cytometer (Beckman Coulter). At the excitation wavelength of 488 nm, light scattering and red fluorescence were detected. Different cell lines adjusted different parameters.

### 2.11 Wound‐healing migration assay

Subsequent to cell digestion, a single‐cell suspension was established and deployed for seeding into 6‐well culture plates. Plates were scratched manually after a 24 h incubation. The scratched culture plate was gently washed with PBS to remove scratched cells, serum‐free medium was utilized, and pictures were taken at 0 h. Afterward, the culture plate was put into the incubator again for 24 h. Using a microscope, the peripheral cells were checked whether they have migrated to the central scratch area, and pictures were taken.

### 2.12 Transwell assay

Tumor cells from different groups of distinct cell lines were extracted and resuspended in serum‐free medium. Thereafter, the cells were counted, and the resultant cell density was calibrated to 1–10 × 10^5^/mL. Following this, 200 μL of the refined cell suspension was added to the top chamber while taking keen precautions to avoid air bubbles as much as possible. Subsequent to the addition of serum‐free medium containing the tumor cells into the upper chamber, 500 μL of complete medium augmented with fetal bovine serum was supplemented to the lower well. The resultant plate was thereafter placed inside the incubator for 48 h. As soon as the incubation was completed, the cells situated in the lower chamber were duly fixed in precooled 10% formaldehyde and stained using 0.1% crystal violet. Finally, cell enumeration was conducted under a microscope.

### 2.13 Xenograft experiments in vivo

Twenty‐four healthy Balb/c nude female mice of specific pathogen‐free level (weight, 20 g) were selected for the experiment. The Animal Experiment Center of Jilin University provided nude mice. Throughout the experiment, all nude mice were consistently maintained under standard conditions, with the ambient temperature being 22 ± 2°C and the relative humidity remaining around 50% ± 5%, artificially controlled diurnal cycle lighting for 12 h, kept in separate cages, and given full‐price nutritional feed. No differences in other exposure factors, such as age, and body weight, were found among the groups. The operators involved in the animal experimentation conducted at the First Hospital of Jilin University had all duly cleared the qualification examination for experimental animals and performed the procedures within a strictly controlled environment. The experimental methodology for all animal studies incorporated in this study had been granted ethical clearance by the Animal Experiment Ethics Committee of the First Hospital of Jilin University (20210270). Different groups of A549 tumor cells (2 × 10^6^ cells) were mixed with Matrigel and injected subcutaneously into the middle and outer sides of the right axilla of nude mice. The skin of the nude mice was disinfected before injection. The body weight of nude mice and subcutaneous tumor size were detected every 7 days. On Day 28, the nude mice were sacrificed. The tumor volume was measured as follows: volume = length × width^2^ × Π/6, and the tumor growth curve was drawn.

### 2.14 Statistical analysis

All data analyses in this experiment were progressed via IBM SPSS Statistics version 19.0 (IBM Corp.). The outcomes of continuous variables have been delineated herein as mean ± standard deviation. Subsequently, comparative analysis between two groups was performed via *t*‐tests, while analysis among multiple groups was conducted through the analysis of variance. The comparison between two groups was conducted by *t*‐tests, and the comparison between multiple groups was conducted by the analysis of variance. Furthermore, categorical variables were evaluated utilizing chi‐square test (*p < 0.05, **p < 0.01, ***p < 0.001).

## 3 RESULTS

### 3.1 High GPR37 expression predicted a poor prognosis through network data analysis

**FIGURE 1:**
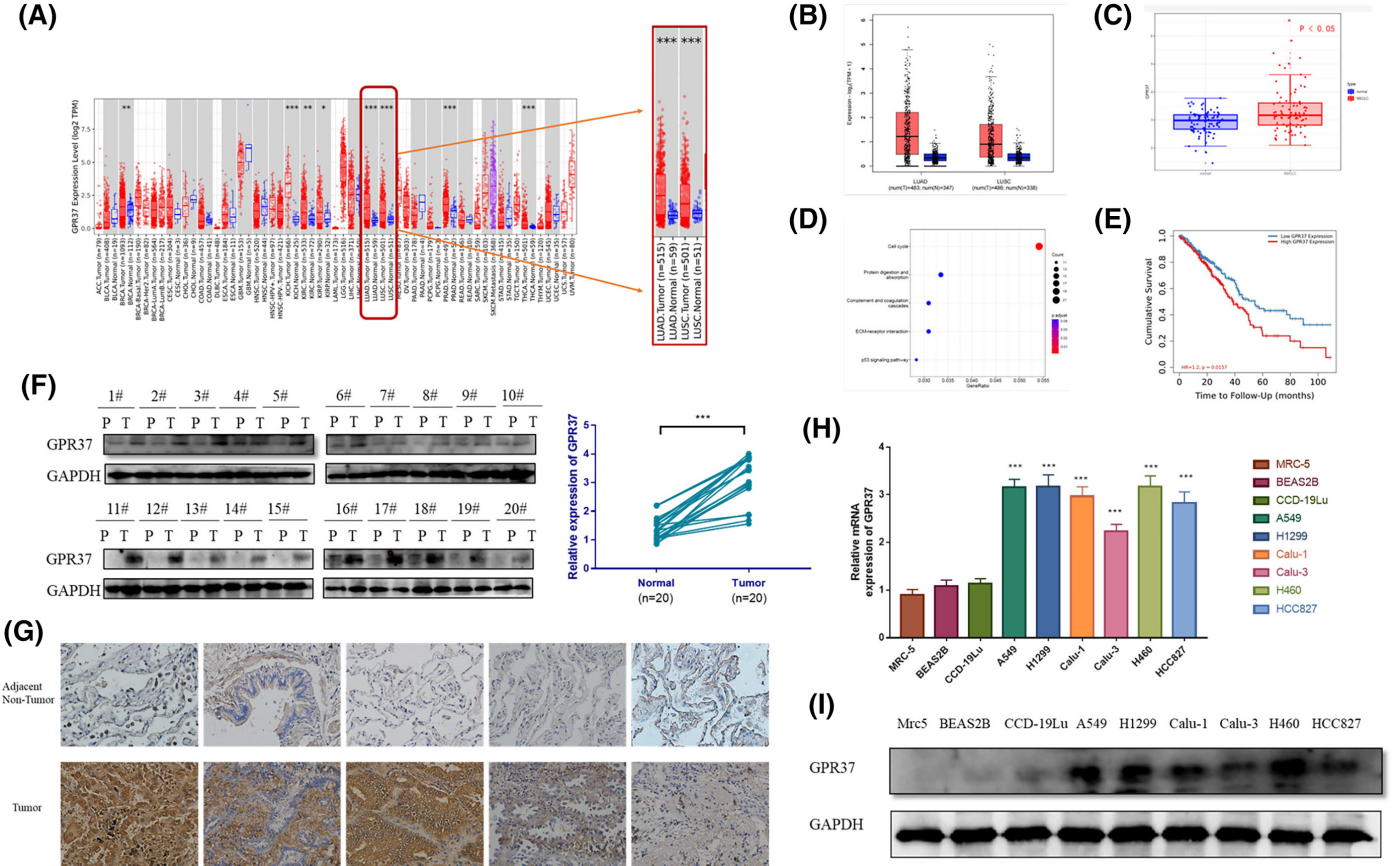
GPR37 was highly expressed in NSCLC cells and tissues predicting poor prognosis. (A) Analysis of the differential expression of GPR37 in LUAD and LUSC using TCGA database data. (B) The expression of GPR37 in LUAD and LUSC tissues through the GEPIA2 online tool (*p* > 0.05). (C) The expression level of GPR37 was significantly increased in NSCLC samples compared with controls through the GEO database (*p* < 0.05). (D) Enrichment analysis of Kyoto Encyclopedia of Genes and Genomes (KEGG) terms for terms for GPR37 co‐expression genes. (E) The cumulative survival between the high expression group and low expression group of GPR37 was obtained by Kaplan–Meier analysis (*p* < 0.05). (F) The expression level of GPR37 in tumor tissues and matched para‐cancerous tissues evaluated by WB and RT‐PCR. P stands for para‐cancerous tissue, and T stands for tumor tissue. (G) The expression level of GPR37 in tumor tissues and matched para‐cancerous tissues evaluated by IHC. (H) The expression level of GPR37 detected by RT‐PCR in NSCLC cell lines and normal human lung tissue cell lines. (I) The expression level of GPR37 in NSCLC cell lines and normal human lung tissue cell lines detected by WB. *p < 0.05, **p < 0.01, ***p < 0.001.

To seek the expression of GPR37 between normal tissues and different tumors, TCGA database was used, and the online analysis tool of TIMER 2.0 was applied to input GPR37 in the Cancer Exploration module. Notably, the expressions of GPR37 in LUAD and LUSC were markedly elevated compared to the control group (*p* < 0.001). The outcomes of this study have been graphically depicted in Figure 1A.

The GEPIA2 online tool was used to select lung cancer patient information from Genotype‐Tissue Expression (GTEx) and TCGA databases, and differences in GPR37 expression were analyzed in LUAD and LUSC tissues and normal tissues. In 483 LUAD tissues and 347 normal tissues analyzed, it was observed that the expression of GPR37 was indeed amplified in the LUAD tissues. Among the analyzed 338 normal tissues and 486 LUSC tissues, it was discovered that the expression of GPR37 was notably enhanced in the LUSC tissues. In contrast, the analysis showed that the expressions of GPR37 in the two types of lung cancers were not remarkably divergent from those detected in the control group (*p* > 0.05). The outcomes of this analysis have been graphically presented in Figure 1B.

We selected three NSCLC datasets (GSE18842, GSE74706, GSE21933) from the GEO database to study the differential expression of GPR37 in NSCLC samples and normal samples. The three data sets obtained 85 NSCLC samples and 84 control samples. Data analysis revealed that the expression level of GPR37 was significantly increased in NSCLC samples compared with controls (Figure 1C). KEGG pathway analysis demonstrated that the co‐expression of GPR37 was primarily associated to the cell cycle, protein digestion and absorption, complement and coagulation cascades (Figure 1D).

The Survival Analysis module of GEPIA2 was used to evaluate the OS and disease‐free survival (DFS) of GPR37. Participants were divided into the high and low GPR37 expression groups. Patients with LUAD and LUSC in TCGA database were selected for statistical analysis.

Evaluating the OS, the findings showed that the high GPR37 expression group exhibited a notably inferior prognosis in contrast to the low GPR37 expression group, displaying significant statistical significance (*p* < 0.05). In addition, no remarkable difference was detected in DFS between the two groups (*p* > 0.05). These results are exhibited in Figure 1E.

### 3.2 Validation of clinical data and cytology

The demographic characteristics of the patients are shown in Table 1. RNA was extracted from paired para‐cancerous tissues and tumor tissues of 20 patients. Further qRT‐PCR detection was conducted. The expression levels of GPR37 in the tumor tissues were found to be significantly elevated as compared to their corresponding adjacent para‐cancerous lung tissues (*p* < 0.001) (Figure 1F). Moreover, proteins were extracted from the tumor tissues and paired para‐cancerous tissues of these 20 patients, and Western blot (WB) detection was performed. The results corresponded with RT‐PCR findings, where the expression levels of GPR37 within tumor tissues were notably elevated in comparison with their para‐cancerous tissue counterparts, with statistically significant differences (*p* < 0.001). Both tumor and para‐cancerous tissue samples were additionally subjected to IHC analyses, which confirmed a significant difference in GPR37 expression levels between NSCLC and para‐cancerous tissues (*p* < 0.05). A detailed outline of the examination results is available in Table 2. Schematic representations of IHC outcomes for some tumor tissues and adjacent para‐cancerous tissues are visualized in Figure 1G.

**TABLE 1:** The demographic characteristics of the patients.

	Patients (*N* = 20)
Age, years	
Mean (SD)	53.05 (11.85)
Median	52.5
Q1, Q3	46.5, 63.5
Range	31–73
Sex	
Male	12 (60%)
Female	8 (40%)
Type of pathology	
Squamous carcinoma	9 (45%)
Adenocarcinoma	8 (40%)
Large cell carcinoma	2 (10%)
Adenosquamous carcinoma	1 (5%)
Stage of lung cancer	
IA	9 (45%)
IB	6 (30%)
IIA	5 (25%)

**TABLE 2:** The expression level of GPR37.

	Negative	Weak	Moderate	Strong positive	Total	*p* value
NSCLC	1	7	10	2	20	<0.05
Para‐cancerous tissues	13	7	0	0	20	

Abbreviations: GPR37, G protein‐coupled receptor 37; NSLCL, non‐small‐cell lung cancer.

Three normal human lung tissue cell lines (Mrc5, BEAS2B, and CCD‐19Lu) were selected as the control group, and different NSCLC cell lines (A549, H1299, Calu‐1, Calu‐3, H460, and HCC827) were used to verify GPR37 expression. Through RT‐PCR and WB detection, the expressions of GPR37 in normal human lung cells and tumor cells were observed, as shown in Figure 1H,I. GPR37 was slightly expressed in normal human lung tissue cell lines, and no major statistical differences in GPR37 expressions were accessed (*p* > 0.05). The results of the BEAS2B cell line, which was utilized as the control group in the statistical analysis, were conducted to evaluate the expression levels. However, the expressions of GPR37 in the six NSCLC cell lines were considerably greater than those observed in the BEAS2B cell line, with highly statistical differences (*p* < 0.001). We chose H1299, H460, and A549 cell lines with higher expression for subsequent experiments. By WB detection, the expression levels of Mrc5, BEAS2B, and CCD‐19Lu were very low in normal human lung tissue cells; however, the expression of GPR37 was notably amplified in NSCLC cell lines (*p* < 0.001).

### 3.3 Construction of stable knockdown GPR37 cell lines

**FIGURE 2:**
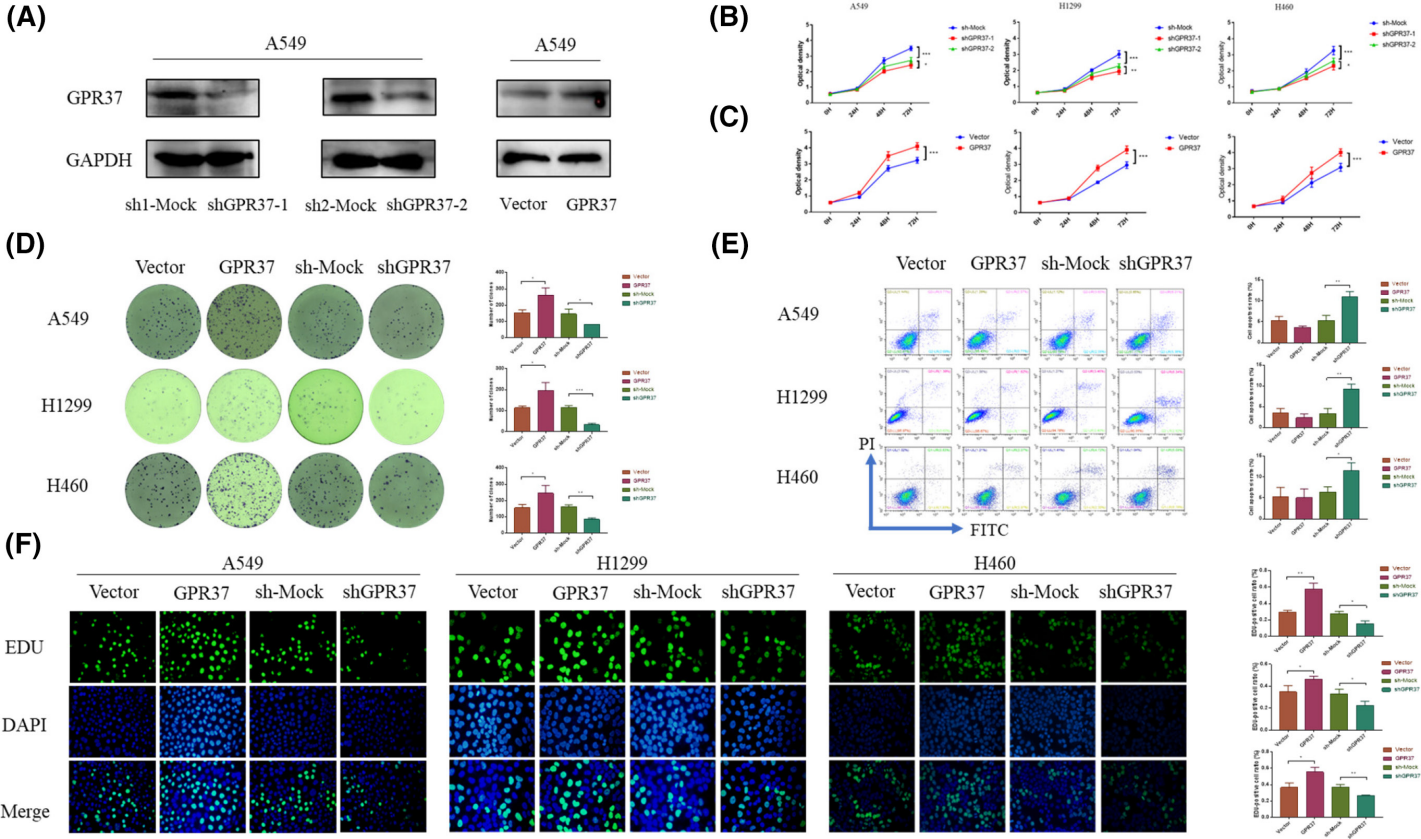
Construction of stable GPR37 knockout cell line and the effect of GPR37 on the viability, apoptosis, and proliferation of NSCLC cells. (A) The expression of GPR37 in two stably knocked down A549 cell lines by WB. (B) Evaluations of the influence of shGPR37‐1, shGPR37‐2, and sh‐Mock groups on the cell viability by CCK‐8 assay. (C) Evaluations of the influence of GPR37 on cell viability of different groups for knockdown and control group by CCK‐8 assay. (D) Evaluations of the influence of GPR37 on cell proliferation ability of different groups by colony formation assay. (E) Evaluations of the influence of GPR37 on apoptosis in NSCLC cells by FACS in different groups. (F) Evaluations of the influence of GPR37 on DNA synthesis ability by EdU assay. *p < 0.05, **p < 0.01, ***p < 0.001.

Two A549 cell lines with stable knockdown of GPR37 were successfully constructed. After 10 passages, the GPR37 knockdown efficiency was detected by WB (Figure 2A). WB detection showed that after transfection of A549 cell lines with different GPR37 knockdown lentiviral packaging plasmids, the expression of GPR37 was different, that is, the endogenous expression of the target *GPR37* gene had a knockdown effect. In addition, the expression level in the shGPR37‐1 cell line is more inhibited than that in the shGPR37‐2 cell line, considering that the knockdown efficiency of shGPR37‐1 is better.

To further verify the expression efficiency of shGPR37‐1 and shGPR37‐2, three cell lines including H1299, H460, and A549 were constructed, and the corresponding GPR37 knockdown negative control group (sh‐mock group) was created. Through CCK‐8 experiment, the absorbance with time under the 450 nm wavelength light of the microplate reader was compared (Figure 2B). After 72 h of culture, the cell viability rate in the GPR37 knockdown group was notably lower than that in the sh‐mock group (*p* < 0.001). In H1299, H460, and A549 cell lines, the cell viability rate was lower in the shGPR37‐1 group than in the shGPR37‐2 group. Statistically remarkable differences were detected amid the three groups (*p* < 0.05, *p* < 0.01, and *p* < 0.05). The findings corresponded to the results in Figure 2A, considering that the expression level of the shGPR37‐1 cell line was more inhibited than that of the shGPR37‐2 cell line. As a result, we selected the stably transfected cell lines after shGPR37‐1 transfection as the subsequent GPR37 knockdown group (shGPR37 group).

### 3.4 GPR37 can promote the cell proliferation ability of NSCLC

Three stably transfected cell lines of A549, H1299, and H460 overexpressing GPR37 (GPR37 group) and a stably transfected cell line transfected with an empty vector packaging plasmid (vector group) were constructed. Through the CCK‐8 experiment, absorbance change times under the 450‐nm wavelength light of the microplate reader were compared (Figure 2C). After 72 h of culture, among the three NSCLC cell lines, the cell viability rate in the GPR37 group was significantly stronger than that in the vector group, with significant differences (*p* < 0.001).

The colony formation ability was detected by a clone formation experiment. The ordinate on the right of the histogram is the number of clones formed, and the abscissa presents the groups analyzed (Figure 2D). It was revealed that the GPR37 group displayed a significantly enhanced cell clone formation ability in comparison with the vector group (*p* < 0.05). Moreover, the cell colony formation potential of the shGPR37 group was notably diminished in contrast to that of the sh‐mock group. In A549, H1299, and H460 cell lines, the results were all statistically significant (*p* < 0.01).

The DNA synthesis ability was detected by the EdU experiment. The vertical axis on the right of the histogram is the ratio of EdU‐positive cells to the total number of cells, and the horizontal axis presents the groups analyzed (Figure 2F). In H1299, H460, and A549 cell lines, it was revealed that a prominent increase in the cell DNA synthesis ability of the GPR37 group compared to the vector group (*p* < 0.01, *p* < 0.05, and *p* < 0.05). Conversely, a notable decrease in DNA synthesis ability was observed in the shGPR37 group concerning the sh‐mock group (*p* < 0.05, *p* < 0.05, and *p* < 0.01).

### 3.5 The GPR37 gene can reduce cell apoptosis to a certain extent

FACS was employed to detect cell apoptosis. H1299, H460, and A549 cell lines were evaluated (Figure 2E). In H1299, H460, and A549 cell lines, there was a slightly lower apoptosis rate in the GPR37 group compared to the vector group, statistical analysis demonstrated no significant difference between the two groups (*p* > 0.05, *p* > 0.05, and *p* > 0.05). The apoptosis rate of the shGPR37 group was remarkably higher than that of the sh‐mock group (*p* < 0.01, *p* < 0.01, and *p* < 0.05).

### 3.6 GPR37 can promote the migration and invasion ability of NSCLC cells

**FIGURE 3:**
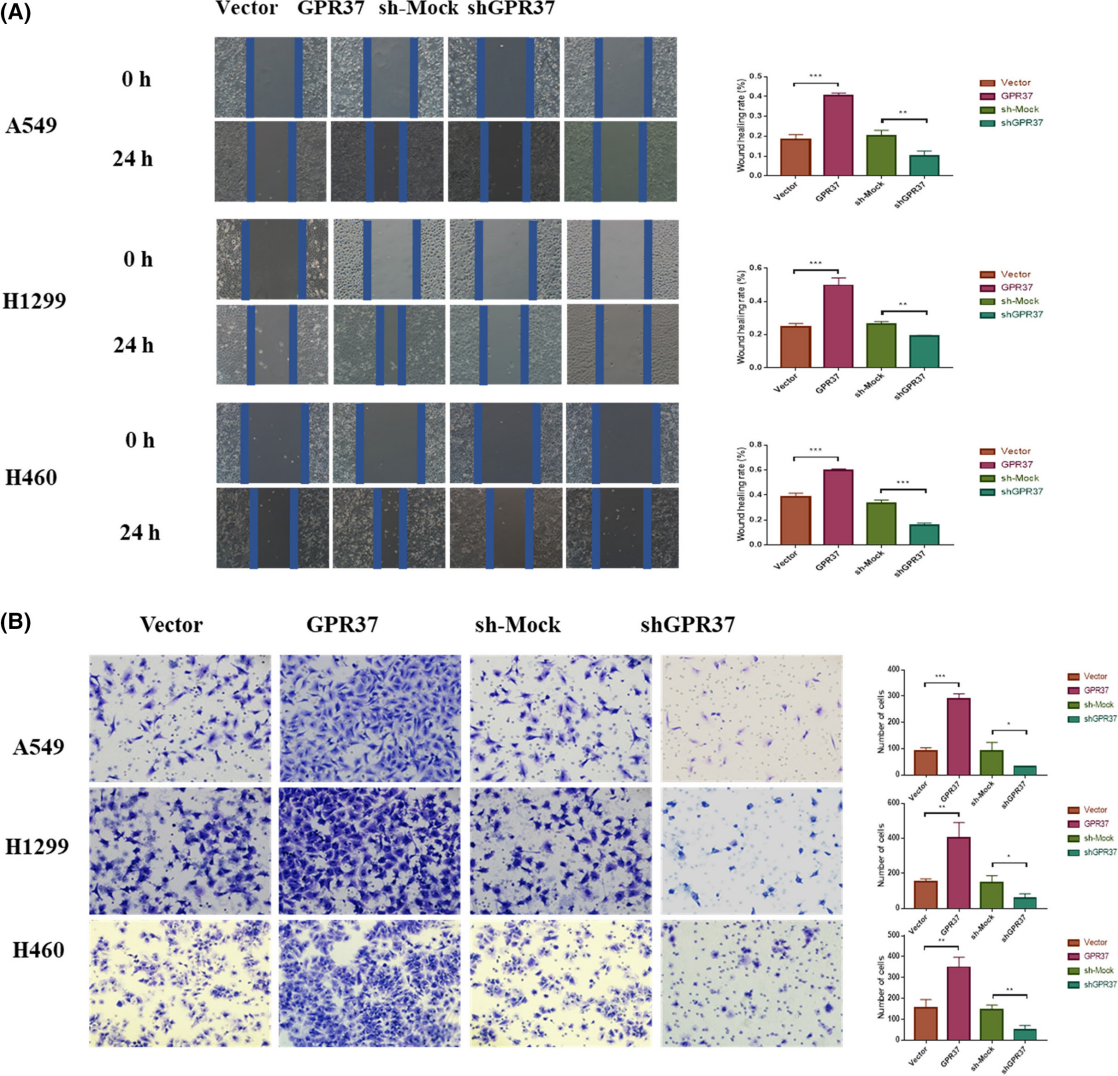
GPR37 can promote the migration and invasion ability of NSCLC cells. (A) Evaluations of the influence of GPR37 on cell migration ability by wound‐healing migration assay. (B) Evaluations of the influence of GPR37 on cell invasiveness by Transwell assay. *p < 0.05, **p < 0.01, ***p < 0.001.

The migration ability of A549, H1299, and H460 cell lines in the GPR37, vector, shGPR37, and sh‐mock groups was observed by the cell scratch test (Figure 3A). In H1299, H460, and A549 cell lines, the cell migration ability of the GPR37 group was notably stronger than that of the vector group, showing statistical differences (*p* < 0.001, *p* < 0.001, and *p* < 0.001). The cell migration rate of the shGPR37 group was considerably weaker than that of the sh‐mock group, showing statistical differences (*p* < 0.01, *p* < 0.01, and *p* < 0.001).

The cell invasion ability was evaluated via the Transwell experiment (Figure 3B). In H1299, H460, and A549 cell lines, the number of cells passing through Matrigel and filter membrane in the GPR37 group was remarkably higher than that in the vector group (*p* < 0.001, *p* < 0.01, and *p* < 0.01). The cell migration rate of the shGPR37 group was weaker than that of the sh‐mock group, showing statistical differences (*p* < 0.05, *p* < 0.05, and *p* < 0.01).

### 3.7 GPR37 can induce drug resistance in NSCLC

**FIGURE 4:**
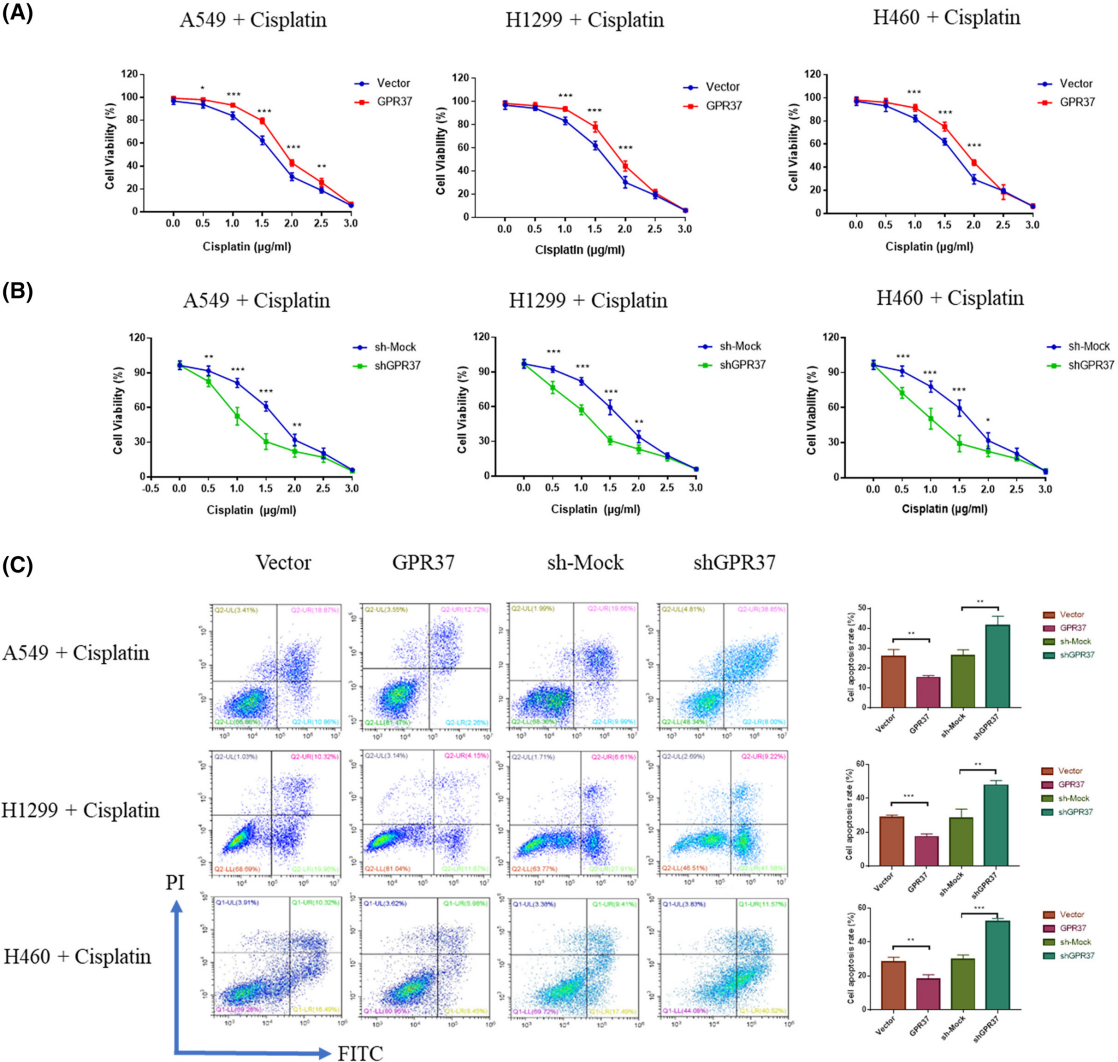
GPR37 can induce drug resistance in NSCLC. (A) The cell viability of GPR37 group and Vector group after adding different concentrations of cisplatin was detected by CCK‐8 assay. (B) The cell viability of shGPR37 group and sh‐Mock group after adding different concentrations of cisplatin was detected by CCK‐8 assay. (C) Evaluations of the influence of GPR37 on apoptosis in NSCLC cells by FACS after adding cisplatin. *p < 0.05, **p < 0.01, ***p < 0.001.

Gradient concentrations of cisplatin (0, 0.5, 1.0, 1.5, 2.0, 2.5, and 3.0 μg/mL) were added and cultivated for 24 h. The CCK‐8 experiment was conducted to explore the influence of GPR37 on tumor cell regulation in the context of cisplatin sensitivity, as displayed in Figure 4A. In the A549 cell line, the cell viability of the GPR37 group began to be higher than that of the control group when the cisplatin concentration was 0.5 μg/mL, and the difference was notably significant (*p* < 0.05). As the concentration increased, the difference remained significant and persisted (*p* < 0.001, *p* < 0.001, *p* < 0.001, and *p* < 0.01) until the concentration reached 3.0 μg/mL; no statistical difference was noted in the cell viability between the two groups (*p* > 0.05). Meanwhile, H1299 and H460 cell lines obtained similar results.

After adding different concentrations of cisplatin, changes in the cell viability in the sh‐mock and shGPR37 groups were analyzed (Figure 4B). In the A549 cell line, a significant decline in cell viability was initiated at the 0.5 μg/mL cisplatin concentration, relative to the control group (*p* < 0.01). As the concentration increased, the difference was still significant and persisted (*p* < 0.001, *p* < 0.001, and *p* < 0.01). No statistical difference in cell viability was detected between the two groups until the concentration reached 2.5 μg/mL (*p* > 0.05). The results for H460 and H1299 cell lines trended similarly to those of the A549 cell line.

H1299, H460, and A549 cell lines in the logarithmic growth phase were selected in different groups. Cisplatin at a concentration of 1.0 μg/mL was added for 24 h. The effect of GPR37 on the regulation of tumor cell sensitivity to cisplatin was observed by FACS (Figure 4C). In the A549 cell line, the apoptosis rate of the GPR37 group was lower than that of the vector group (*p* < 0.01), whereas the apoptosis rate of the shGPR37 group was higher than that of the sh‐mock group (*p* < 0.01). The results for the H460 and H1299 cell lines trended similarly to those for the A549 cell line. In the FACS detection, the H1299 and H460 cell apoptosis levels in different groups under the action of cisplatin were similar to that of the A549 cell line.

### 3.8 GPR37 can promote the tumorigenic ability and growth rate of NSCLC in vivo

**FIGURE 5:**
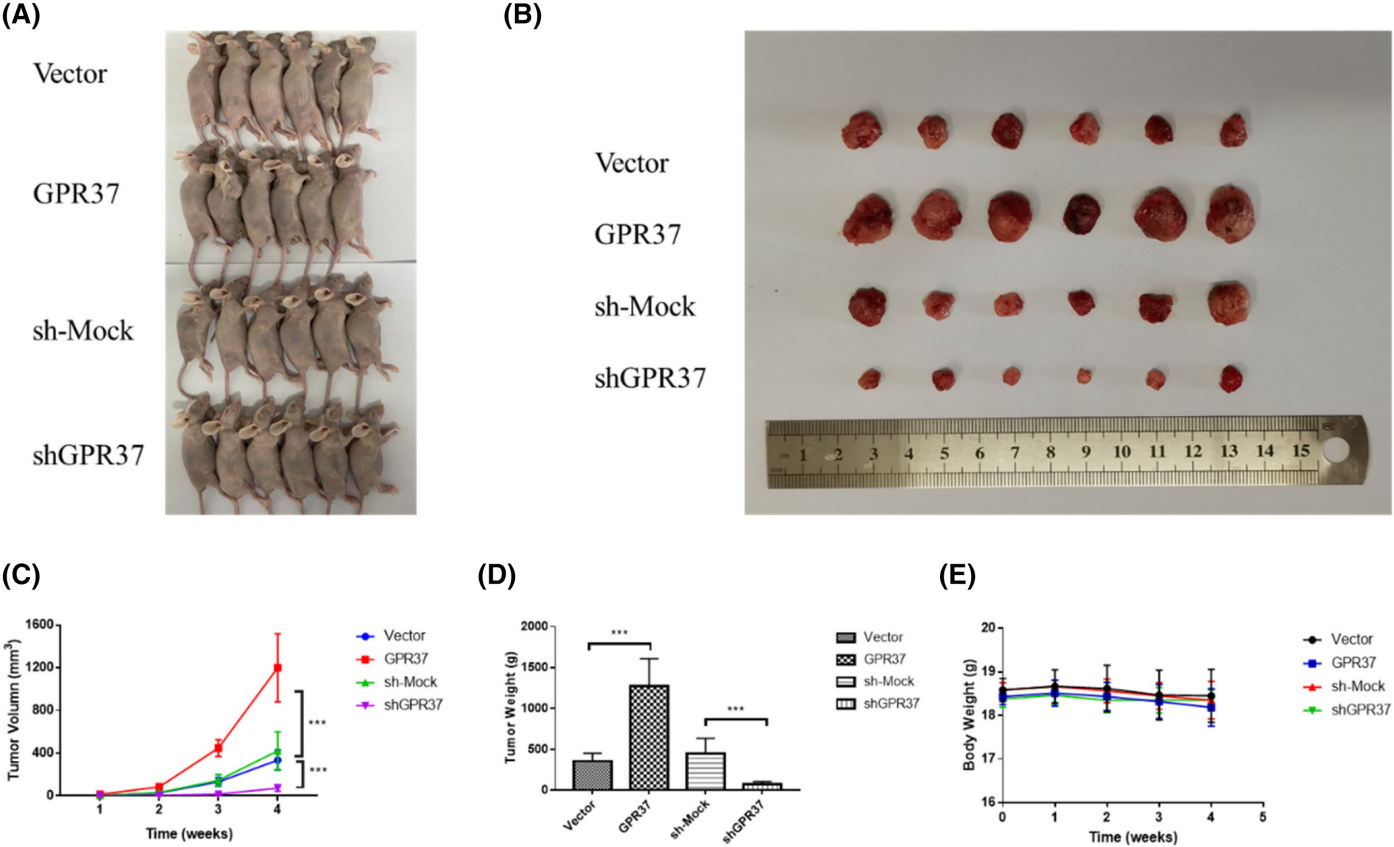
GPR37 can promote the tumorigenic ability and growth rate of NSCLC in vivo. (A) Nude mice 28 days after subcutaneous injection of tumor cells. (B) Subcutaneous tumors were dissected from nude mice. (C) Changes in tumor volume over time. (D) Histogram of tumor weight. (E) Trend of body weight of nude mice. ***p < 0.001.

To evaluate the effect of GPR37 on NSCLC growth in animals, 24 randomly selected female nude mice were separated into four groups, as described in the methodology. The A549 cells in the vector, GPR37, sh‐mock group, and shGPR37 groups were treated and injected subcutaneously into the middle and outer sides of the right axilla of nude mice. The volume of the subcutaneous tumor was calculated every 7 days after injection, and the body weight of the nude mice was measured every 7 days until Day 28 (Figure 5). Figure 5A–C presents images of nude mice after subcutaneous injection of tumor cells at 28 days, subcutaneous tumors that have been stripped out, and the change in tumor volume, respectively. The tumor volume and weight of the GPR37 group were higher than those of the vector group (*p* < 0.001). The tumor volume and weight of the shGPR37 group were both lower than those of the sh‐mock group, and the difference was statistically significant (*p* < 0.001). Figure 5D,E presents the tumor weight and the trend of bodyweight of nude mice, respectively. The bodyweight of the nude mice did not decrease.

### 3.9 GPR37 is involved in EMT

**FIGURE 6:**
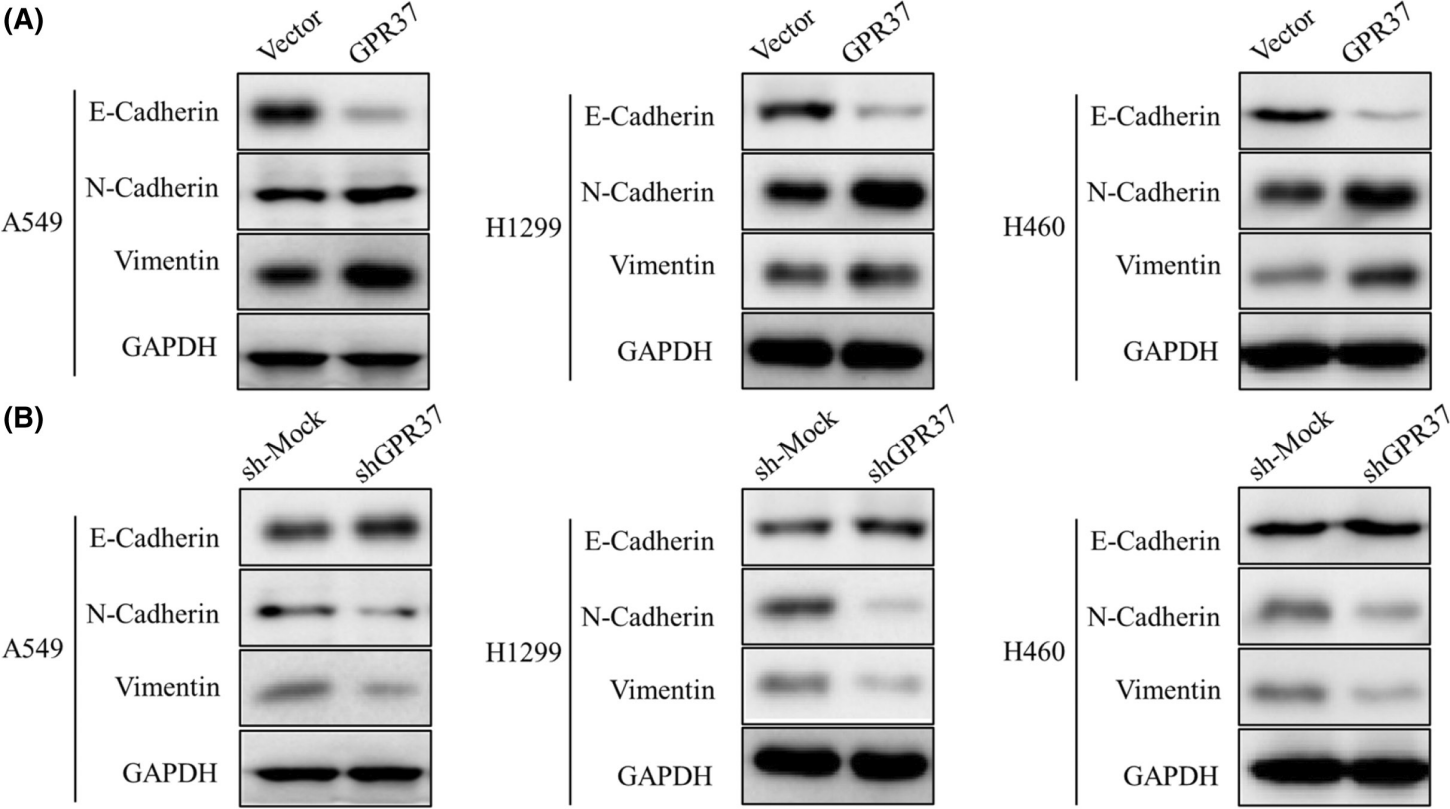
GPR37 is involved in the occurrence of EMT in NSCLC. (A) Expressions of EMT‐related markers with GPR37 overexpression in NSCLC by WB. (B) Expressions of EMT‐related markers with GPR37 downregulation in NSCLC by WB.

To verify the effect of GPR37 on EMT in NSCLC, through WB experiments, the expressions of vimentin, N‐cadherin, E‐cadherin, and three biomarkers related to EMT were detected (Figure 6). As shown in Figure 6A, the expression of E‐cadherin was downregulated in the GPR37 group compared to the vector group, while the expressions of N‐cadherin and vimentin were markedly upregulated in the same group, in the A549 cell line analysis. The results were similar to those of the H1299 and H460 cell lines. This shows that GPR37 promotes EMT.

### 3.10 GPR37 regulates the PI3K/Akt/mTOR pathway and promotes NSCLC progression

**FIGURE 7:**
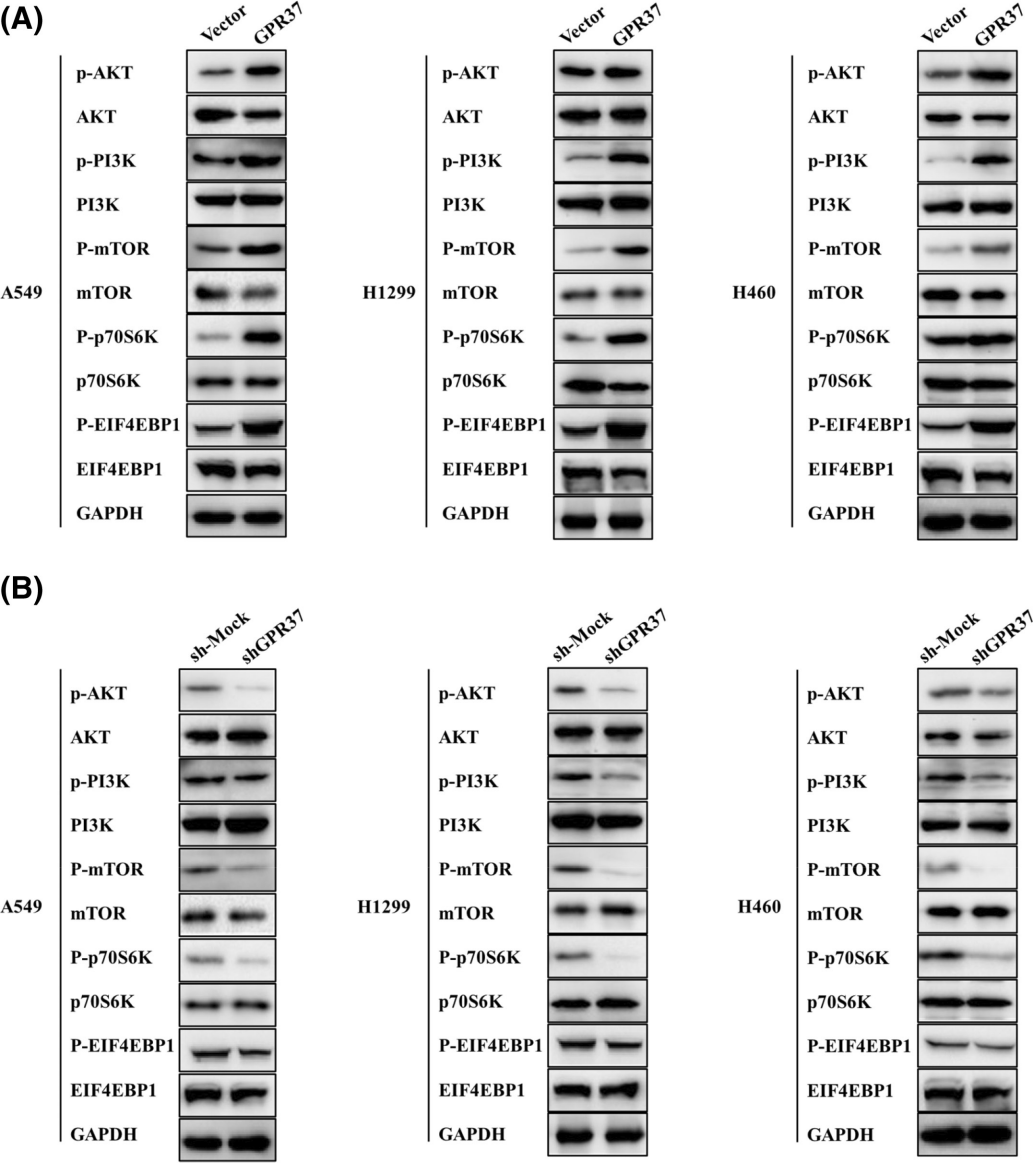
GPR37 regulates the PI3K/Akt/mTOR pathway and promotes the progression of NSCLC. (A) Expressions of PI3K/Akt/Mtor‐pathway‐related markers with GPR37 overexpression in NSCLC by WB. (B) Expressions of PI3K/Akt/Mtor‐pathway‐related markers with GPR37 downregulation in NSCLC by WB.

To verify that GPR37 mediates the progression of NSCLC via the PI3K/Akt/mTOR pathway, proteins closely related to the PI3K/Akt/mTOR pathway, such as P‐AKT1, AKT1, EIF4EBP1, P‐EIF4EBP1, P‐p70S6K, p70S6K, mTOR, P‐mTOR, PI3K, and P‐PI3K were detected. In these three cell lines, the expressions of P‐EIF4EBP1, P‐p70S6K, P‐mTOR, P‐PI3K, and P‐AKT were significantly raised in the GPR37 group, indicating that GPR37 overexpression can trigger the PI3K/Akt/mTOR signal transduction pathway (Figure 7A).

Meanwhile, we measured the key proteins of the above signal transduction pathway by WB of the shGPR37 and sh‐mock groups. In these three cell lines, the expressions of P‐EIF4EBP1, P‐p70S6K, P‐mTOR, P‐PI3K, and P‐AKT were significantly declined in the shGPR37 group (Figure 7B). This showed that GPR37 knockdown can inhibit the PI3K/Akt/mTOR signal transduction pathway and tumor occurrence and development. To ensure the reliability and reproducibility of our results, all experiments were performed at least three times.

## 4 DISCUSSION

According to the data provided by the WHO in 2021, although the incidence of lung cancer has been relegated as the second largest malignant tumor worldwide, the number of new cases is still increasing annually. Unfortunately, the 5‐year survival rate for NSCLC is still approximately 20%.^31^ Even with the survival rate increase in targeted drug therapy, disease prognosis remains unsatisfactory.

GPCR encodes at least 800 human genes, including seven transmembrane proteins, which regulate important physiological processes through the synergy of signaling pathways.^32^

Our study also involved a screening of a public database, where we discovered a significant raise in GPR37 expression in NSCLC patients compared to the healthy population (*p* < 0.001). In addition, the high expression of GPR37 was shown to predict poor prognosis in NSCLC patients (*p* < 0.05), suggesting that GPR37 could pose as an independent prognostic indicator for NSCLC. These findings highlight the potential clinical utility of GPR37 in guiding personalized cancer treatment and outcomes for NSCLC patients. Subsequently, through clinical verification, the expressions of GPR37 in different tumor tissues were remarkably higher than that in paired para‐cancerous tissues (*p* < 0.001). However, because the follow‐up period of the patients was <5 years, no further analysis of the OS was performed. Related work still needs to be further improved. Through cell experiments, we found that GPR37 was upregulated in NSCLC cell lines, which verified the important role of GPR37 in NSCLC. Studies have demonstrated that GPR37 is highly expressed in NSCLC and impacts the prognosis, suggesting its important role in NSCLC progression.

With more than 800 members, GPCRs are the largest family of cell surface molecules responsible for signal transmission, accounting for nearly 2% of the human genome codes. Moreover, it is estimated that approximately 50%–60% of current therapeutic drugs act either directly or indirectly on GPCRs, reflecting their essential role in human physiology and disease. Dysfunctions in GPCR signaling have been implicated in numerous pathologies, ranging from cardiovascular diseases and metabolic disorders to neuropsychiatric and immune‐related disorders, making them attractive targets for drug development. Recent experimental and clinical data have shown^33^, ^34^, ^35^, ^36^ that GPCRs play important but not fully understood roles in cancer progression and metastasis. Essentially, we have known that malignant cells can manipulate normal physiological functions of GPCRs to drive malignant progression and metastasis. By hijacking these receptors signaling pathways, cancer cells can proliferate autonomously, evade immune surveillance, enhance oxygen and nutrient delivery, invade surrounding tissues, and spread to distant areas. They have been identified as drivers of unregulated growth in certain endocrine tumors. These mutations can lead to the constitutive activation of GPCRs, even when there are no ligands present to activate them. Additionally, some human oncogenic DNA genomes have been shown to express GPCRs, further highlighting their role in cancer development. However, aberrant GPCR overexpression by agonists released by tumor cells or stromal cells and their autocrine and paracrine activation are the most used strategies for tumor cells to stimulate GPCRs and their signaling networks. In addition, GPCRs have emerged as crucial targets of inflammatory mediators, providing a possible link between cancer development and chronic inflammation. Dysregulation of GPCR signaling has been involved in various processes central to tumor progression, including tumor‐induced angiogenesis and metastasis. Indeed, GPCR‐guided migration has been shown to facilitate the spread of cancer cells to target organs, highlighting the importance of GPCRs in cancer metastasis. Therefore, targeting GPCRs and their downstream effectors represents a promising avenue for developing novel mechanism‐based strategies for cancer prevention, diagnosis, and treatment. As a member of the GPCR family, GPR37 is of particular interest as a potential therapeutic target. In this study, by constructing GPR37 overexpression, and knockdown lentiviral packaging plasmids and transfecting H1299, H460, and A549 cells, cell lines with stable expression were obtained. The effect of GPR37 on cell function was explored. The results revealed that the upregulated of the *GPR37* gene can modulate the invasion, migration, and proliferation of NSCLC cells, inhibit cell apoptosis, and increase the resistance to chemotherapy drugs. Knocked down *GPR37* gene can reduce the invasion, migration, and proliferation ability of NSCLC cells, induce apoptosis, and increase the sensitivity to chemotherapy drugs, which plays a potential therapeutic role and might be a novel target for NSCLC treatment. Moreover, in vivo experiments, overexpressed GPR37 can promote tumorigenesis and growth of NSCLC in animals, whereas knocked down GPR37 can inhibit tumorigenesis and growth of NSCLC in animals. This conclusion is similar to Wang's and Xie's studies,^15^, ^16^ but is different from Li's research, namely that low levels of GPR37 are associated with tumor progression and poorer patient survival,^13^ so further validation is needed. Indicated that GPR37 may exhibit different mechanisms in different cancers.

After tumor cells undergo EMT, they not only have stronger invasiveness but also are more likely to develop resistance to anti‐tumor drugs. When epidermal growth factor (EGF) and transforming growth factor Beta (TGF‐β) lead to EMT, they can also induce drug resistance of NSCLC cells to cisplatin and paclitaxel.^37^ In verifying the effect of GPR37 on the EMT of NSCLC, we found that the results of different cell lines were similar, that is, the expression of E‐cadherin in the GPR37 group was lower than that in the vector group, whereas the expressions of vimentin and N‐cadherin were upregulated. This shows that GPR37 promotes the occurrence of EMT. Moreover, GPR37 can lead to the transition of cell morphology to mesenchymal‐like cells, promote cell migration, invasion, and metastasis, and at the same time make tumor cells more resistant to drugs, which is consistent with the previous outcomes of this study.

Dysregulated PI3K/Akt pathway is oncogenic in some cases. The crucial role of dysregulated PI3K activity in cancer onset and progression has been extensively documented. For instance, the PI3K p110 subunit gene *PI3KCA* frequently mutates in human cancers,^26^ and PI3K is a major growth factor activation pathway in LNCaP human prostate cancer cells.^38^ Other studies have revealed the important role of PI3K activity in a variety of tumors, such as leukemia,^39^ lung cancer,^40^ and breast cancer.^41^ In this study, the expression levels of P‐EIF4EBP1, P‐p70S6K, P‐mTOR, P‐PI3K, and P‐AKT in the GPR37 group were considerably higher than those in the vector group. Thus, GPR37 can activate key proteins in the PI3K/Akt/mTOR pathway and promote its activation. GPR37 knockdown can inhibit Raf/MEK/ERK and PI3K/Akt/mTOR activation. The inhibition of NSCLC progression, thus playing a potential therapeutic role, may become a new target in NSCLC treatment.

This study also has shortcomings. The number of clinical verification samples was too small. The different expression levels and prognosis of GPR37 were not analyzed clinically. This study lacks in‐depth animal studies to verify the mechanism of GPR37 in promoting NSCLC development. The novelty of this study is related to its evaluation of the involvement of GPR37 in NSCLC. GPR37 knockdown was found to act a tumor‐suppressor role in NSCLC and could be used as a potential therapeutic target in NSCLC. This study also confirmed for the first time that GPR37 can promote the EMT of NSCLC and GPR37 can activate the PI3K/Akt/mTOR signal transduction pathway to promote NSCLC progression. This study elucidated the expression changes of a variety of downstream genes caused by the high GPR37 expression in NSCLC at the molecular level, pointing out the molecular mechanism of accelerating the proliferation of NSCLC cells, inhibiting cell apoptosis, increasing cell migration and invasion ability, and increasing the probability of drug resistance. This new idea of GPR37 as an important regulatory gene was proposed and might be a novel target in the therapy of NSCLC.

## AUTHOR CONTRIBUTIONS

**Han Liu:** Funding acquisition (equal); methodology (equal); project administration (equal); writing – original draft (equal). **Yingjie Zhu:** Methodology (equal). **Huikun niu:** Data curation (equal); formal analysis (equal). **Jing Jie:** Data curation (equal). **Shucheng Hua:** Conceptualization (equal); investigation (equal). **Xiaoxue Bai:** Methodology (equal). **Shuai Wang:** Conceptualization (equal); writing – review and editing (equal). **Lei Song:** Conceptualization (equal); writing – review and editing (equal).

## FUNDING INFORMATION

This work was supported by Science and Technology Development Program Project of Jilin Provincial Department of Science and Technology (YDZJ202201ZYTS108) and the Science and Technology Development Project of Changchun Science and Technology Bureau (21ZGM20).

## CONFLICT OF INTEREST STATEMENT

The authors confirm that the study was performed without any financial or commercial relationships that might be perceived as potential conflicts of interest.

## ETHICS STATEMENT

The research method of animal experiments was endorsed by the Animal Experiment Ethics Committee of the First Hospital of Jilin University (20210270). All patients signed the informed consent. The trial was endorsed by the Ethics Committee of the First Hospital of Jilin University (2021–170).

## Data Availability

The data utilized and analyzed in this study are available upon reasonable request from the corresponding author, in accordance with the data‐sharing policies of the journal.
